# MicroRNA miR-509 Regulates ERK1/2, the Vimentin Network, and Focal Adhesions by Targeting Plk1

**DOI:** 10.1038/s41598-018-30895-8

**Published:** 2018-08-22

**Authors:** Guoning Liao, Ruping Wang, Alyssa C. Rezey, Brennan D. Gerlach, Dale D. Tang

**Affiliations:** Department of Molecular and Cellular Physiology, Albany Medical College, Albany, New York USA

## Abstract

Polo-like kinase 1 (Plk1) has been implicated in mitosis, cytokinesis, and proliferation. The mechanisms that regulate Plk1 expression remain to be elucidated. It is reported that miR-100 targets Plk1 in certain cancer cells. Here, treatment with miR-100 did not affect Plk1 protein expression in human airway smooth muscle cells. In contrast, treatment with miR-509 inhibited the expression of Plk1 in airway smooth muscle cells. Exposure to miR-509 inhibitor enhanced Plk1 expression in cells. Introduction of miR-509 reduced luciferase activity of a Plk1 3′UTR reporter. Mutation of miR-509 targeting sequence in Plk1 3′UTR resisted the reduction of the luciferase activity. Furthermore, miR-509 inhibited the PDGF-induced phosphorylation of MEK1/2 and ERK1/2, and cell proliferation without affecting the expression of c-Abl, a tyrosine kinase implicated in cell proliferation. Moreover, we unexpectedly found that vimentin filaments contacted paxillin-positive focal adhesions. miR-509 exposure inhibited vimentin phosphorylation at Ser-56, vimentin network reorganization, focal adhesion formation, and cell migration. The effects of miR-509 on ERK1/2 and vimentin were diminished in RNAi-resistant Plk1 expressing cells treated with miR-509. Taken together, these findings unveil previously unknown mechanisms that miR-509 regulates ERK1/2 and proliferation by targeting Plk1. miR-509 controls vimentin cytoskeleton reorganization, focal adhesion assembly, and cell migration through Plk1.

## Introduction

Smooth muscle cell proliferation and migration play a pivotal role in regulating development and homeostasis of internal organs, and contribute to the progression of many pathological processes such as airway remodeling^1–4^. The mechanisms that regulate smooth muscle cell proliferation and motility are not fully understood. Polo-like kinase 1 (Plk1) is a serine/threonine protein kinase that has been implicated in mitosis and cytokinesis^5,6^. In addition, Plk1 regulates the proliferation of various cell types including smooth muscle cells^7^ and cancer cells^8^. Plk1 modulates smooth muscle cell proliferation by controlling the phosphorylation of MEK1/2 and ERK1/2 in response to activation of growth factors^7,9^. Moreover, c-Abl (Abelson tyrosine kinase, Abl) participates in the regulation of smooth muscle cell proliferation^9–11^.

The intermediate filament protein vimentin is also associated with the pathogenesis of smooth muscle diseases including vascular remodeling in cardiovascular illness^12^. The vimentin network has been shown to modulate nonmuscle cell migration^13,14^. Vimentin intermediate filaments may regulate cell migration by affecting microtubule regrowth and actin cytoskeletal reorganization in the leading edge^13,15^. More importantly, vimentin undergoes phosphorylation at Ser-56, which has been implicated in regulating cancer cell invasion and migration^16–18^. In smooth muscle, Plk1 catalyzes vimentin phosphorylation at Ser-56^19^ whereas vimentin dephosphorylation at this position is mediated by type 1 protein phosphatase^20^.

MicroRNAs (miRNAs) are a class of small noncoding RNAs (18–25 nucleotides) that posttranscriptionally regulate the expression of target genes and regulate a variety of cellular processes^21,22^. In general, miRNAs bind to complementary sequences in the 3′ untranslated regions (3′UTR) of target mRNAs, which may lead to target mRNA degradation and/or translational repression^21,22^. miR-100 has been reported to target Plk1 in cancer cells including liver cancer cells^23^ and nasopharyngeal cancer cells^24^. On the other hand, miR-203 regulates expression of c-Abl tyrosine kinase and smooth muscle cell proliferation^25^. miR-25 is involved in regulation of Kruppel-like factor 4 and phenotype of smooth muscle cells^26^. However, the nature of miRs that regulate Plk1 expression in smooth muscle cells remains to be elucidated.

In this study, we unexpectedly find that miR-100 does not regulate Plk1 expression in human airway smooth muscle cells. In contrast, hsa-miR-509-3-5p (miR-509) controls Plk1 expression in smooth muscle cells. miR-509 regulates ERK1/2 and proliferation via Plk1. Moreover, miR-509 modulates the vimentin network, focal adhesions, and cell migration.

## Results

### miR-100 Does Not Affect Plk1 Expression in Human Airway Smooth Muscle Cells

**Figure 1:**
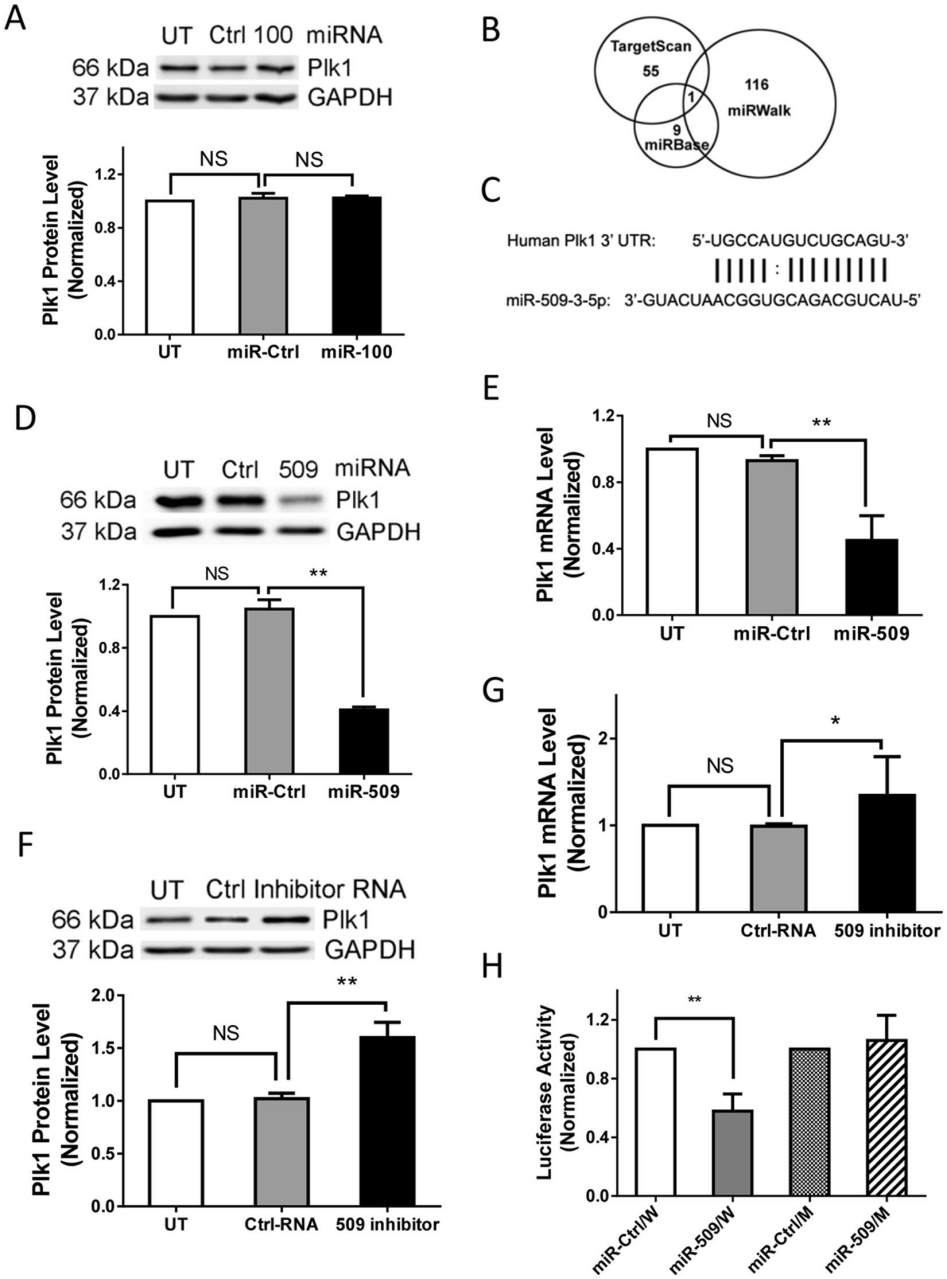
Plk1 is regulated by miR-509 at mRNA and protein levels in smooth muscle cells. (**A**) Human airway smooth muscle (HASM) cells were transfected with either 20 nM miR-control (miR-Ctrl) or miR-100, or they were untransfected, for 3 days. Blots of the HASM cells were probed with antibodies against Plk1 and glyceraldehyde-3-phosphate dehydrogenase (GAPDH). Data are mean ± SD (n = 4). NS, not significant. (**B**) All three online miR search tools predict 3′UTR of human Plk1 as a target of miR-509. (**C**) Sequence alignment between miR-509 and 3′UTR of human Plk1. (**D**) Blots of untransfected HASM cells and cells transfected with either miR-Ctrl or miR-509 for 3 days were probed with antibodies against Plk1 and GAPDH. Data are mean ± SD (n = 4, **p < 0.01). (**E**) HASM cells were transfected with either 20 nM miR-Ctrl or miR-509, or they were untransfected for 3 days. Plk1 mRNA in the cells was assessed by RT-qPCR (reverse transcription quantitative real-time polymerase chain reaction). Data are mean ± SD (n = 4, **p < 0.01). (**F**) HASM cells were untransfected, or transfected with either 20 nM control RNA or miR-509 inhibitor for 2 days. miR-509 inhibitor is small, chemically modified single-strand RNA molecules designed to specifically bind to and inhibit endogenous miR-509. Blots of the cells were probed with antibodies against Plk1 and GAPDH. Data are mean ± SD (n = 4, **p < 0.01). (**G**) mRNA of Plk1 in untransfected cells and cells transfected with either 20 nM control RNA or miR-509 inhibitor for 2 days was assessed by RT-qPCR. Data are mean ± SD (n = 7, *p < 0.05). UT, untransfected cells. (**H**) Relative luciferase activity in cells cotransfected with plasmids encoding either wild-type (W) or mutant (M) Plk1 3′UTR plus miR-Ctrl or miR-509. Data are mean ± SD (n = 5, **p < 0.01). One-way ANOVA was used for statistical analysis.

Because miR-100 has been reported to target Plk1 in cancer cells^23,24^, we evaluated the role of miR-100 in regulating Plk1 in smooth muscle cells. Human airway smooth muscle (HASM) cells were transfected with either miR-control or miR-100 mimics for 3 days. Immunoblot analysis was used to assess protein expression. Treatment with miR-control did not significantly affect the expression of Plk1 (Fig. 1A, n = 4, one-way ANOVA test). More importantly, we unexpectedly found that treatment with miR-100 did not significantly reduce Plk1 protein level in smooth muscle cells (Fig. 1A, n = 4, one-way ANOVA test). The results imply that miR-100 dependent regulation of Plk1 is cell-type specific.

### miR-509 Regulates Plk1 Expression in Smooth Muscle Cells

We used miRNA analysis online tools (TargetScanHuman, miRbase, and miRwalk) to search for other miRs that may regulate Plk1. We found that 55 miRs from TargetScanHuman, 116 miRs from miRWalk and 9 miRs from miRbase likely target 3′UTR of human Plk1 mRNA (NCBI accession number: NM_005030.5). Moreover, all these three online tools predict that hsa-miR-509-3-5p (miR-509) targets Plk1 3′UTR (Fig. 1B,C). To assess the role of miR-509, HASM cells were transfected with either miR-control or miR-509 mimics for 3 days. Protein levels of Plk1 evaluated by immunoblot analysis were reduced by approximately 60% in cells transfected with miR-509 (Fig. 1D, n = 4, *p* < 0.01, one-way ANOVA test). Plk1 mRNA levels assessed by RT-qPCR were significantly reduced in cells treated with miR-509 by approximately 50% (Fig. 1E, n = 4, *p* < 0.01, one-way ANOVA test). Because Plk1 is critical for mitosis and proliferation, complete knockdown of Plk1 can impede cell viability. Thus, we used the cell model, in which Plk1 was partially downregulated, but significantly, for this study.

To evaluate whether endogenous miR-509 affects Plk1 expression, cells were treated with either control RNA or miR-509 inhibitor (also known as antimiR). miR-509 inhibitor is small and chemically modified single-strand RNA molecules designed to specifically bind to and inhibit endogenous miR-509. Exposure to miR-509 inhibitor enhanced Plk1 expression at protein (Fig. 1F, n = 4, *p* < 0.01, one-way ANOVA test) and mRNA levels (Fig. 1G, n = 7, *p* < 0.05, one-way ANOVA test). These results suggest that miR-509 regulates Plk1 expression in smooth muscle cells.

### miR-509 Targets 3′UTR of Plk1 mRNA

To determine whether miR-509 targets 3′UTR of Plk1 mRNA, we generated mutant 3′UTR of Plk1 mRNA, in which 6 nucleotides were replaced with mismatched bases (Fig. S1A). HEK 293 cells were transfected with wild type or mutant Plk1 3′UTR reporter (Fig. S1B) plus miR-control or miR-509 for 2 days. Treatment with miR-509 reduced the luciferase activity of wild type Plk1 3′UTR reporter (Fig. 1H, n = 5, *p* < 0.01, one-way ANOVA test). However, the luciferase activity of mutant 3′UTR reporter was not diminished in cells treated with miR-509. Moreover, miR-control did not affect the luciferase activity of wild type and mutant 3′UTR reporters (Fig. 1H, n = 5, not significant, one-way ANOVA test).

### Expression of miR-509 Is Reduced in Asthmatic HASM Cells

**Figure 2:**
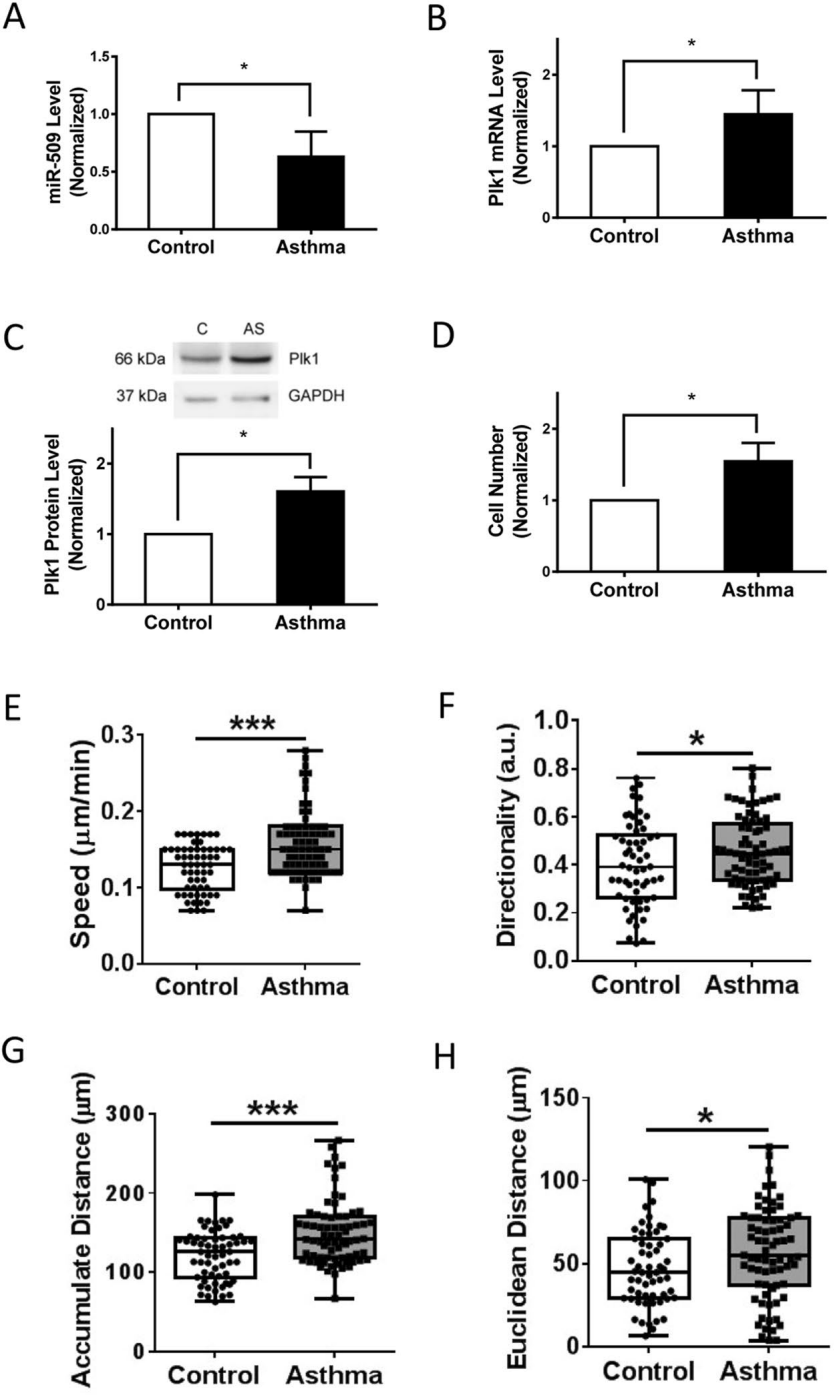
Asthmatic human airway smooth muscle cells display reduced miR-509 expression, increased Plk1 expression, and enhanced proliferation and migration. (**A**) miR-509 expression in control and asthmatic HASM cells were determined using the experimental procedure described in Materials and Methods. miR-509 expression is reduced in asthmatic smooth muscle cells (Control cells from 5 donors, asthmatic cells from 4 donors, *p < 0.05). However, expression of Plk1 at mRNA (**B**) and protein (**C**) levels is higher in asthmatic HASM cells (Control cells from 5 donors, asthmatic cells from 4 donors, *p < 0.05). (**D**) Control and asthmatic HASM cells were treated with 10 ng/ml PDGF for 3 days followed by cell counting. The proliferation of asthmatic HASM cells is increased as compared to control cells (Control cells from 5 donors, asthmatic cells from 4 donors, *p < 0.05). (**E**–**H**). Control and asthmatic HASM cells were replated in 6-well dishes for 5 hours, and migration of these cells was then evaluated by time-lapse microscopy for additional 16 h. NIH ImageJ software was used to analyze cell migration speed, directionality, accumulated distance, and Euclidean distance (*p < 0.05; ***p < 0.01; control cells, n = 58 from 5 donors; asthmatic cells, n = 72 from 4 donors). Student’s *t*-test was used for statistical analysis.

Asthma is characterized by airway remodeling, which is largely attributed to smooth muscle growth and motility^2,3,9,27–29^. Because miR-509 affects Plk1 expression, this prompted us to assess its expression in asthmatic HASM cells. miR-509 expression was reduced in asthmatic HASM cells (Fig. 2A, control = 5, asthma = 4, *p* < 0.05, t-test). In contrast, Plk1 mRNA was increased in asthmatic HASM cells (Fig. 2B, control = 5, asthma = 4, *p* < 0.05, t-test). Moreover, Plk1 protein expression was higher in asthmatic HASM cells (Fig. 2C, control = 5, asthma = 4, *p* < 0.05, t-test).

We also compared the proliferation and migration of non-asthmatic (control) vs. asthmatic HASM cells. Asthmatic HASM cells proliferated faster than control cells (Fig. 2D, control = 5, asthma = 4, *p < 0.05, t-test), which was supported by previous findings by others^30^. Moreover, migration of control and asthmatic HASM cells was evaluated by time-lapse microscopy. NIH ImageJ software was used to quantify migratory properties of cells. Accumulated distance, Euclidean distance, speed, and directionality of asthmatic HASM cells were enhanced compared to control HASM cells (Fig. 2E–H, (58 control cells from 5 donors, 72 asthmatic cells from 4 donors, p < 0.05 or 0.01, t-test). These results suggest that reduced miR-509 expression is associated with enhanced Plk1 expression in asthmatic HASM cells. Asthmatic HASM cells display enhanced proliferative and migratory features.

### Treatment with miR-509 Inhibits Cell Proliferation

**Figure 3:**
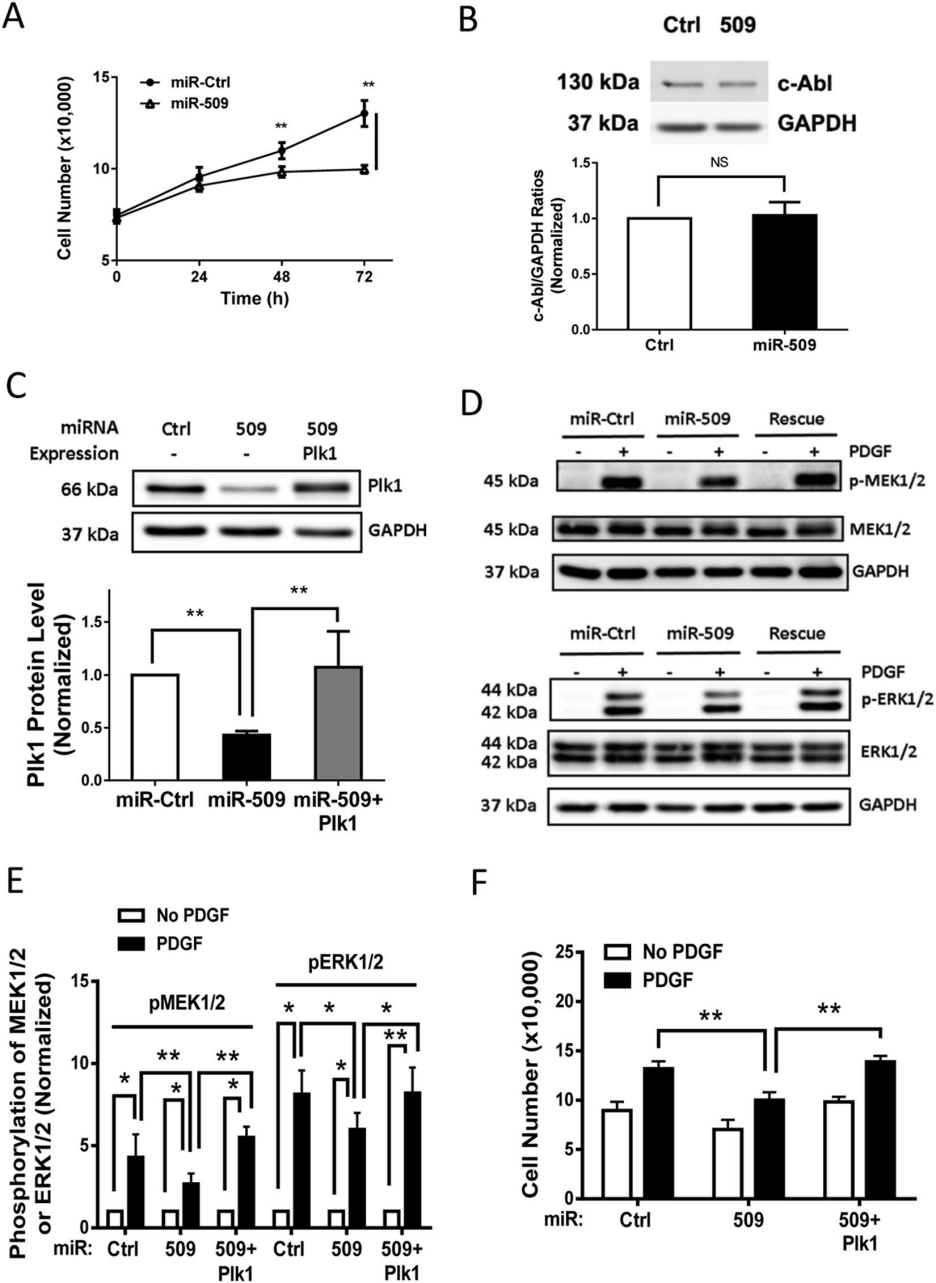
Role of miR-509 in cell proliferation, c-Abl expression, MEK1/2 and ERK1/2 phosphorylation. (**A**) HASM cells were transfected with miR-Ctrl or miR-509. One day after transfection, they were stimulated with 10 ng/ml PDGF for additional 1–3 days followed by assessment of cell numbers. Exposure to miR-509 inhibits the PDGF-induced proliferation. Data are mean ± SD (n = 5, **p < 0.01). (**B**) Treatment with miR-509 does not affect c-Abl protein expression. Blots of HASM cells transfected with either miR-Ctrl or miR-509 for 3 days were probed with antibodies against c-Abl and GAPDH. Data are mean ± SD (n = 5). NS, not significant. (**C**) HASM cells treated with miR-Ctrl or miR-509 for 3 days, or stable Plk1 (miR-509 resistant) expressing cells were treated with miR-509 for 3 days (rescue cells). Cell extracts were evaluated by immunoblot analysis. Data are mean ± SD (n = 5, **p < 0.01). (**D**) Representative immunoblots illustrating the role of Plk1 in phosphorylation of MEK1/2 (MEK1: Ser-218/Ser-222; MEK2: Ser-222/Ser-226) and ERK1/2 (ERK1: Thr-202/Tyr-204; ERK2: Thr-185/Tyr-187). HASM cells treated with miR-Ctrl or miR-509, or rescue cells (see above) were stimulated with 10 ng/ml PDGF for 10 min or left unstimulated. MEK1/2 and ERK1/2 phosphorylation in these cells was evaluated by immunoblot analysis. Rescue of Plk1 in miR-509 treated cells restores the PDGF-induced phosphorylation of MEK1/2 and ERK1/2. (**E**) The phosphorylation levels of MEK1/2 and ERK1/2 in cells stimulated with PDGF are normalized to corresponding unstimulated levels. Data are mean ± SD (n = 7, *p < 0.05; **p < 0.01). (**F**) Smooth muscle cells were treated with miR-Ctrl or miR-509, or stable Plk1 expressing cells were treated with miR-509. One day after treatment, they were stimulated with 10 ng/ml PDGF for 3 days. The numbers of viable cells were then determined. Rescue of Plk1 in miR-509 treated cells recovers the PDGF-induced proliferation in HASM cells. Data are mean ± SD (n = 5, **p < 0.01). Student’s *t*-test was used for statistical analysis of (**A**,**B**). One-way ANOVA was used for statistical analysis of (**C**). Two-way ANOVA was used for statistical analysis of (**E**,**F**).

We evaluated the role of miR-509 in cell proliferation. Cells were transfected with miR-Ctrl or miR-509. One day after transfection, they were stimulated with PDGF for 1–3 days. The numbers of viable cells were then determined. Exposure to miR-509 inhibited the increase of cell number two days after PDGF stimulation (Fig. 3A, n = 5, *p* < 0.01, t-test).

Since c-Abl is involved in the proliferation of various cell types including smooth muscle cells and fibroblasts^9,10,25,31^, we determined the effects of miR-509 on c-Abl expression. Treatment with miR-509 did not affect c-Abl protein expression in cells (Fig. 3B, n = 5, no statistical difference, t-test). The results suggest that miR-509 regulates cell proliferation without affecting c-Abl expression.

### PDGF-induced MAPK Activation Is Regulated by miR-509

Because MAPK signaling is known to regulate cell proliferation^10,11,32^, we evaluated the role of miR-509 in the MAPK pathway. Immunoblot analysis verified downregulation of Plk1 in cells treated with miR-509 (Fig. 3C, n = 5, *p* < 0.01, one-way ANOVA test). More importantly, exposure to miR-509 attenuated the PDGF-induced phosphorylation of MEK1/2 and ERK1/2 as compared to cells treated with miR-control (Fig. 3,D,E, n = 7, *p < *0.05 or 0.01, two-way ANOVA test). Interestingly, PDGF stimulation induced phosphorylation of MEK1/2 and ERK1/2 significantly in cells treated with miR-509 (Fig. 3,D,E, n = 7, *p < *0.05, two-way ANOVA test). The results suggest that miR-509 partially reduces the PDGF-induced phosphorylation of MEK1/2 and ERK1/2.

### RNAi-resistant Plk1 Maintains the PDGF-induced Responses

To further assess the role of Plk1 in miR-509 associated MAPK inhibition, we engineered a novel “rescue” cell model, in which stable Plk1 (resistant to miR-509) expressing cells were treated with miR-509. Immunoblot analysis demonstrated that treatment with miR-509 did not reduce Plk1 expression in the stable Plk1 expressing cells (Fig. 3C, n = 5, *p* < 0.01, one-way ANOVA test), which is similar to a rescue experiment. Phosphorylation of MEK1/2 and ERK1/2 in these cells and cell numbers were then evaluated. RNAi-resistant Plk1 restored the PDGF-induced MEK1/2 and ERK1/2 phosphorylation (Fig. 3,D,E; n = 7, *p < *0.05 or 0.01, two-way ANOVA test) as well as cell proliferation (Fig. 3F, n = 5, *p < *0.01, two-way ANOVA test). These results indicate that miR-509 regulates MEK1/2 and ERK1/2 phosphorylation, and cell proliferation through Plk1.

### miR-509 Regulates Vimentin Filament Organization and Focal Adhesion Assembly

**Figure 4:**
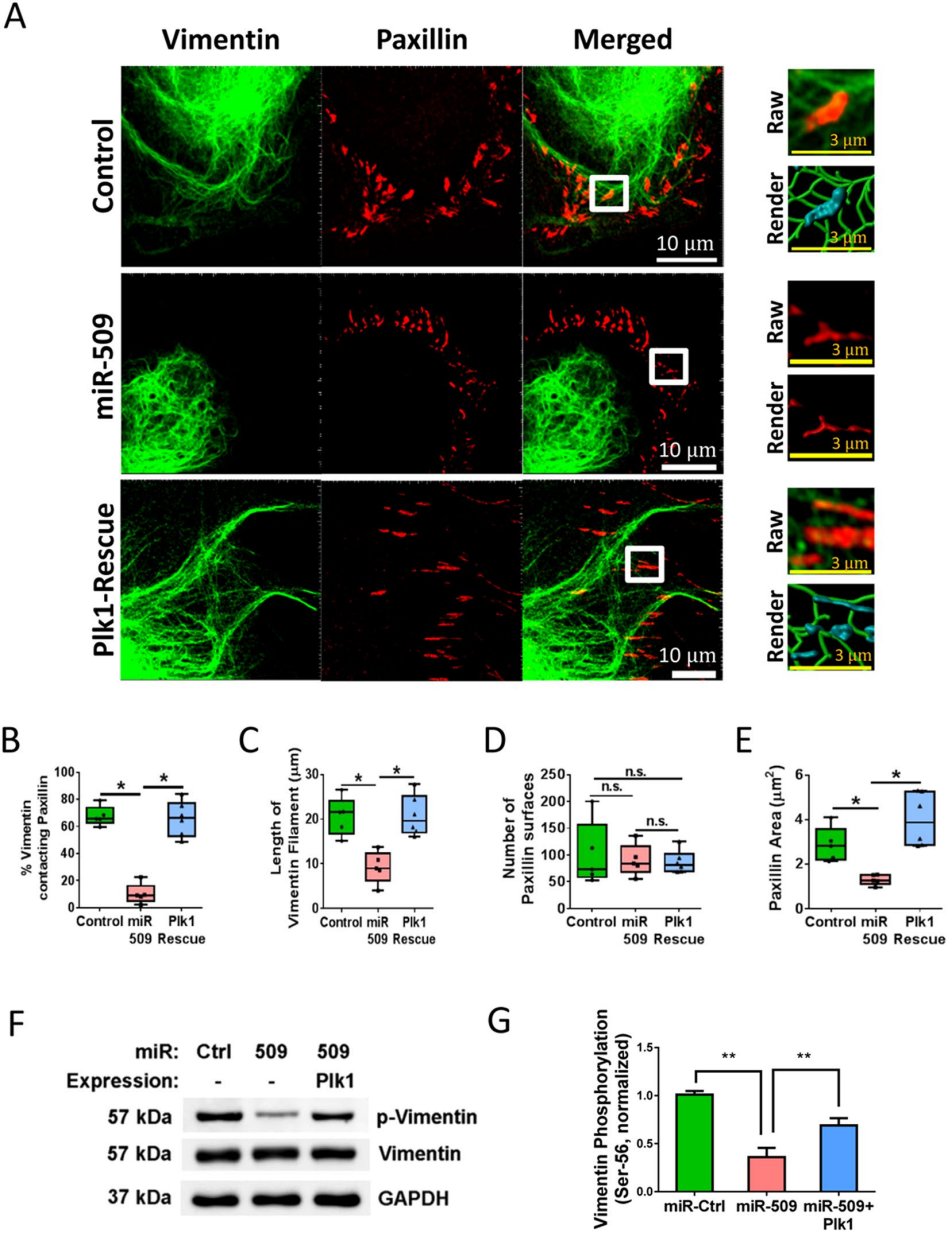
Vimentin network organization and focal adhesion size are regulated by miR-509. (**A**) HASM cells treated with miR-control or miR-509, and rescue cells were stained for vimentin and paxillin. Cell images were taken using a Zeiss LSM880 microscope with Airyscan. The images of vimentin filaments and paxillin staining (>1 µM in length) in cell protrusions were used for Imaris quantitative analysis. White scale bar = 10 μm, yellow scale bar = 3 μm. Imaris software was utilized to 3D-render vimentin filaments and paxillin surfaces. 3D-rendered vimentin is green, paxillin contacting vimentin is cyan, and paxillin alone is red. (**B**) Distance transformation was utilized to quantify the percent of vimentin filaments contacting paxillin surfaces using Imaris software. (**C**–**E**) The length of vimentin filaments, paxillin surfaces and paxillin area were quantified using Imaris software. Statistics used in all graphs were one-way ANOVA with Tukey’s post-hoc test, n = 5, *p < 0.05. (**F**) Representative immunoblots showing the effects of miR-509 and Plk1 on vimentin Ser-56 phosphorylation. Extracts of HASM cells treated with miR-Ctrl, miR-509 or miR-509 plus Plk1 expression construct were immunoblotted with antibodies against phospho-vimentin (Ser-56), total vimentin, and GAPDH. (**G**) Vimentin phosphorylation is normalized to the level obtained from cells treated with miR-Ctrl. Data are mean ± SD (n = 4, **p < 0.01). One-way ANOVA was used for statistical analysis of B–E and G.

Because the vimentin network and focal adhesions are involved in cell migration^16–18,27,33^, HASM cells were immunostained for vimentin and paxillin, a focal adhesion marker^33,34^, and cell images were taken using a confocal microscope. Since cell protrusions are critical for directed migration^27,33^, we chose paxillin-positive focal adhesions (>1 µM in length)^35^ and vimentin filaments in protrusions for analysis. We unexpectedly observed that vimentin intermediate filaments connected with paxillin-positive focal adhesions in cells treated with miR-control (Fig. 4A). We used Imaris software (Bitplane) to quantitatively analyze the confocal microscopic images, and found that approximately 65% of vimentin filaments contacted paxillin in protrusions of control cells (Fig. 4B, n = 5, *p < *0.05, one-way ANOVA test). Treatment with miR-509 reduced connection of vimentin filaments with paxillin, which was restored in rescue cells (Fig. 4A,B, n = 5, *p < *0.05, one-way ANOVA test).

miR-509 also decreased the length of vimentin filaments in protrusions of motile cells (Fig. 4A,C, n = 5, *p < *0.05, one-way ANOVA test). Furthermore, treatment with miR-509 did not affect the number of paxillin surfaces (an indication of paxillin-positive adhesions) (Fig. 4D, n = 5, not significant, one-way ANOVA test). However, miR-509 reduced the area of paxillin of motile cells (Fig. 4E, n = 5, *p < *0.05, one-way ANOVA test). Rescue of Plk1 recovered the length of vimentin filaments and paxillin area (Fig. 4C,E, n = 5, *p < *0.05, one-way ANOVA test). These results suggest that miR-509 regulates the organization of the vimentin network and focal adhesion growth via controlling Plk1 expression.

### miR-509 Treatment Inhibits Vimentin Phosphorylation at Ser-56

Because vimentin Ser-56 phosphorylation has been implicated in vimentin network organization^18,36^, we determined the role of miR-509 in vimentin phosphorylation. Vimentin phosphorylation at Ser-56 was lower in cells treated with miR-509 as compared to control cells; but, recovered by Plk1 rescue (Fig. 4F,G, n = 4, *p < *0.01, one-way ANOVA test).

### Role of miR-509 in Cell Migration

**Figure 5:**
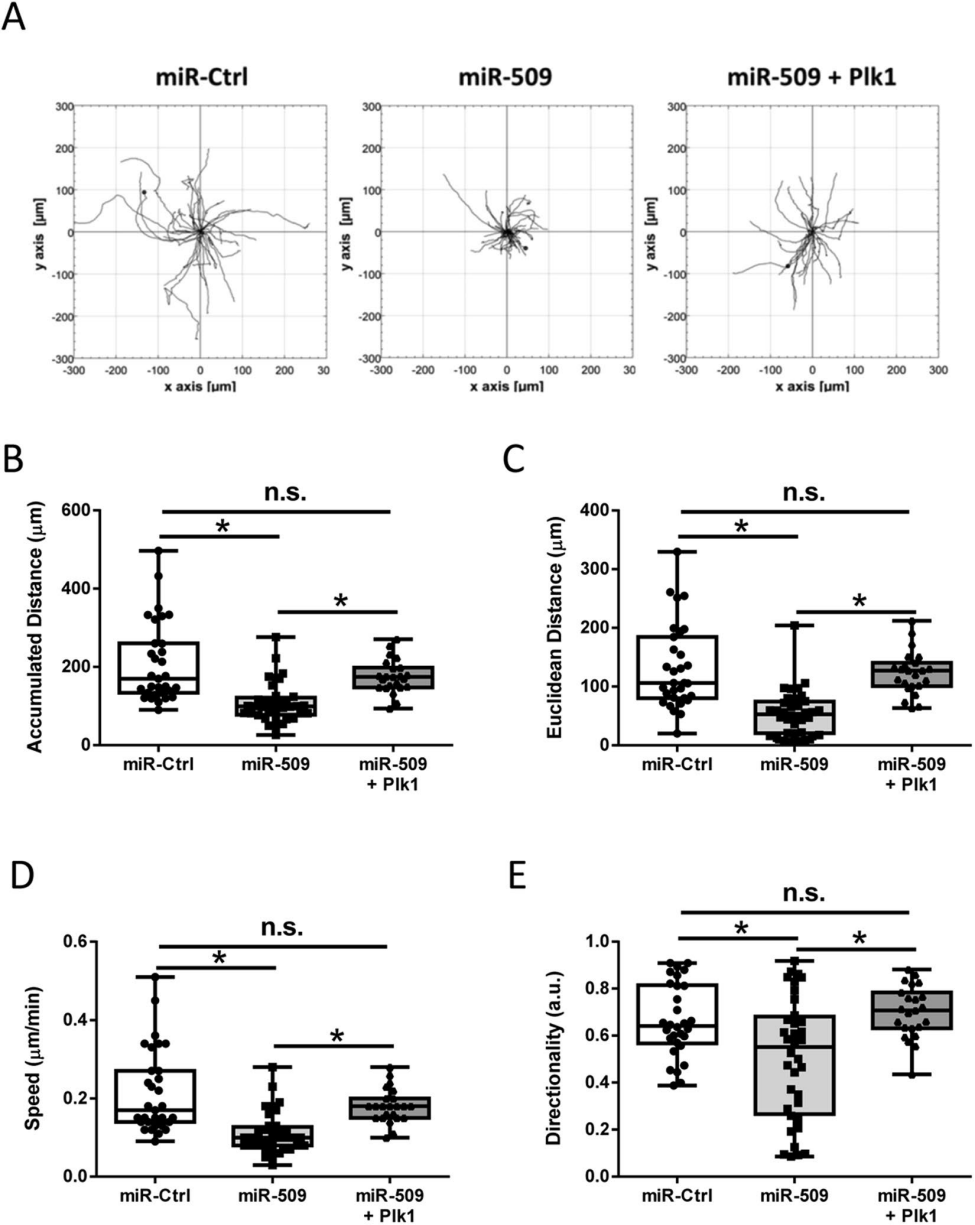
miR-509 negatively regulates smooth muscle cell migration through Plk1. (**A**) Migratory paths of individual cells treated with miR-Ctrl or miR-509, or rescue cells. Treatment with miR-509 reduces accumulated distance (**B**), Euclidean distance (**C**), speed (**D**) and directionality (**E**), which is restored in rescue cells. Data are mean values of 31 miR-Ctrl treated cells, 36 miR-509 treated cells and 23 rescue cells. Error bars indicate SD (*p < 0.05). One-way ANOVA was used for statistical analysis of B–E.

We also evaluated the effects of miR-509 on cell migration. Cells were treated with miR-Ctrl or miR-509 for 3 days. Stable Plk1 expressing cells were treated with miR-509 for 3 days (rescue cells). Cells were then replated in 6-well dishes, and migration of these cells was evaluated using a time-lapse microscope for 16 h. NIH ImageJ software was used to analyze properties of cell migration. Figure 5A showed migration paths of individual cells under these treatments. Exposure to miR-509 reduced accumulated distance (Fig. 5B), Euclidean distance (Fig. 5C), speed (Fig. 5D), and directionality (Fig. 5E) (n = 31 miR-Ctrl treated cells, 36 miR-509 treated cells and 23 rescue cells, *p < 0.05, one-way ANOVA test). Furthermore, Plk1 rescue in miR-509 treated cells restored cell migration (Fig. 5B–E). These results suggest that miR-509 controls smooth muscle migration by affecting Plk1.

## Discussion

Plk1 is a serine/threonine protein kinase that has been implicated in mitosis^5,6^, smooth muscle cell proliferation^7^, and contraction^19^. The role of miRs in regulating Plk1 expression remains to be elucidated. Because miR-100 has been reported to target Plk1 in certain cancer cells^23,24^, we assessed the effects of miR-100 on Plk1 expression in smooth muscle cells. We unexpectedly found that miR-100 treatment did not affect Plk1 expression in this cell type. In contrast, bioinformatics analysis indicated Plk1 as a target of miR-509. In this study, miR-509 reduced Plk1 expression whereas miR-509 inhibitor enhanced Plk1 expression. Furthermore, miR-509 directly targeted the 3′UTR of Plk1 as evidenced by analysis of the luciferase reporter. To the best of our knowledge, this is the first evidence to suggest that miR-509 regulates Plk1 expression by targeting its 3′UTR. In addition, other investigators have found that miR-155 regulation of suppressor of cytokine signaling 1 (SOCS1) is immune cell specific^37^. These results imply that miR regulation of gene expression may be cell-type specific.

The complementary binding of miRNAs to the 3′ UTR of target mRNA may lead to target mRNA degradation and/or translational repression^21,25^. Our current results (Fig. 1D,E) suggest that miR-509 regulates Plk1 expression largely by affecting mRNA degradation. miR-509 may bind to complementary sequence in the 3′ UTR of Plk1, which activates the RNA-induced silencing complex (RISC) and induces Plk1 mRNA degradation^21^. Moreover, our results suggest that endogenous miR-509 may exist in smooth muscle cells, modulating basal Plk1 expression.

In this report, we used Western blotting and RT-qPCR to evaluate the effects of miR-509 inhibitor in cells. We were not able to quantify the levels of miR-509 in cells treated with miR-509 inhibitor (data not shown) because of inconsistent experimental results. This may be because miR-509 inhibitor itself interferes with the measurement of miR-509. Other investigators have shown that miR inhibitors affect the quantification of specific miRs in cells^38,39^.

In this study, asthmatic human airway smooth muscle cells displayed reduced miR-509, higher Plk1 expression, and enhanced proliferation and migration. This prompted us to evaluate the role of miR-509 in cell proliferation and motility. Our results indicate that miR-509 can inhibit cell proliferation and migration. Since miR-509 is reduced in asthmatic airway smooth muscle cells, it is likely that lower miR-509 expression may enhance smooth muscle cell proliferation and migration, which may contribute to the development of airway remodeling in asthma.

Cell proliferation is coordinated by MEK1/2, ERK1/2 and c-Abl tyrosine kinase, important protein kinases implicated in smooth muscle diseases^9–11,31,32^. Our results (Fig. 3) suggest that miR-509 regulates smooth muscle cell proliferation by targeting Plk1, but not c-Abl. The current findings are supported by previous studies that Plk1 affects the MAPK activation^7,9^.

Although miRs selectively regulate their targets, the potential off-target effects of miRs may exist^25,40^. To evaluate the specificity of miR-509, we performed rescue experiments. Traditional rescue experiments are achieved by simultaneous transfection with siRNA (or shRNA) plus plasmids encoding RNAi-resistant gene^41^. Because of relatively low transfection efficiency of plasmids encoding Plk1 in this cell type (data not shown), we constructed recombinant lentivirus for Plk1 expression. Infection of cells with lentivirus expressing miRNA-resistant Plk1 effectively generated stable Plk1 expressing cells. To generate rescue cells, stable Plk1 expressing cells were treated with miR-509. Immunoblot analysis confirmed that the novel approach can maintain Plk1 expression in miR-509 treated cells (Fig. 3C), which is similar to a rescue experiment. The results also imply that the novel alternative approach is useful when traditional rescue approach is not properly working in some cell types.

Our results support the notion that miR-509 modulates vimentin phosphorylation and reorganization by regulating Plk1 expression. This is not surprising because vimentin phosphorylation at Ser-56 has been shown to regulate reorganization of the vimentin network^17,18,27^. Plk1 has also been implicated in vimentin phosphorylation at this residue^19^. Moreover, we unexpectedly found that vimentin filaments contacted with the focal adhesion protein paxillin, and that vimentin may affect focal adhesion assembly. This finding is intriguing because the vimentin network has been thought to affect microtubule regrowth and actin cytoskeletal reorganization in the leading edge of motile cells^13,15^.

We noticed that Plk1 rescue did not completely restore vimentin phosphorylation (Ser-56) (Fig. 4F,G). This is not surprising because vimentin phosphorylation at this residue is also regulated by p21-activated kinase 1 and type 1 protein phosphatase in smooth muscle^17,18,20,42^. The mechanisms by which vimentin phosphorylation at Ser-56 affects focal adhesion assembly are currently unknown. Interestingly, the vimentin network can recruit the Rac-GEF VAV2 to focal adhesions to promote FAK activation and focal adhesion assembly in nonmuscle cells^43^. PKCε-mediated phosphorylation of vimentin increases integrin translocation to the plasma membrane^43,44^. It is likely that phosphorylation at Ser-56 regulates reorganization of the vimentin network, which affects VAV2 activation, integrin recruitment, and focal adhesion formation. Future studies are needed to test the possibility in this cell type.

As discussed earlier, miR-509 regulates ERK1/2, the vimentin network, and focal adhesions by affecting Plk1 in smooth muscle cells. In addition, although miR-155 has hundreds of predicted targets, disruption of the interaction of miR-155 with SOCS1 (a major target of miR-155) is sufficient to alter the behavior of immune cells^37^. Thus, a single miRNA-mRNA axis is able to regulate cellular functions, which may be cell-type specific.

**Figure 6:**
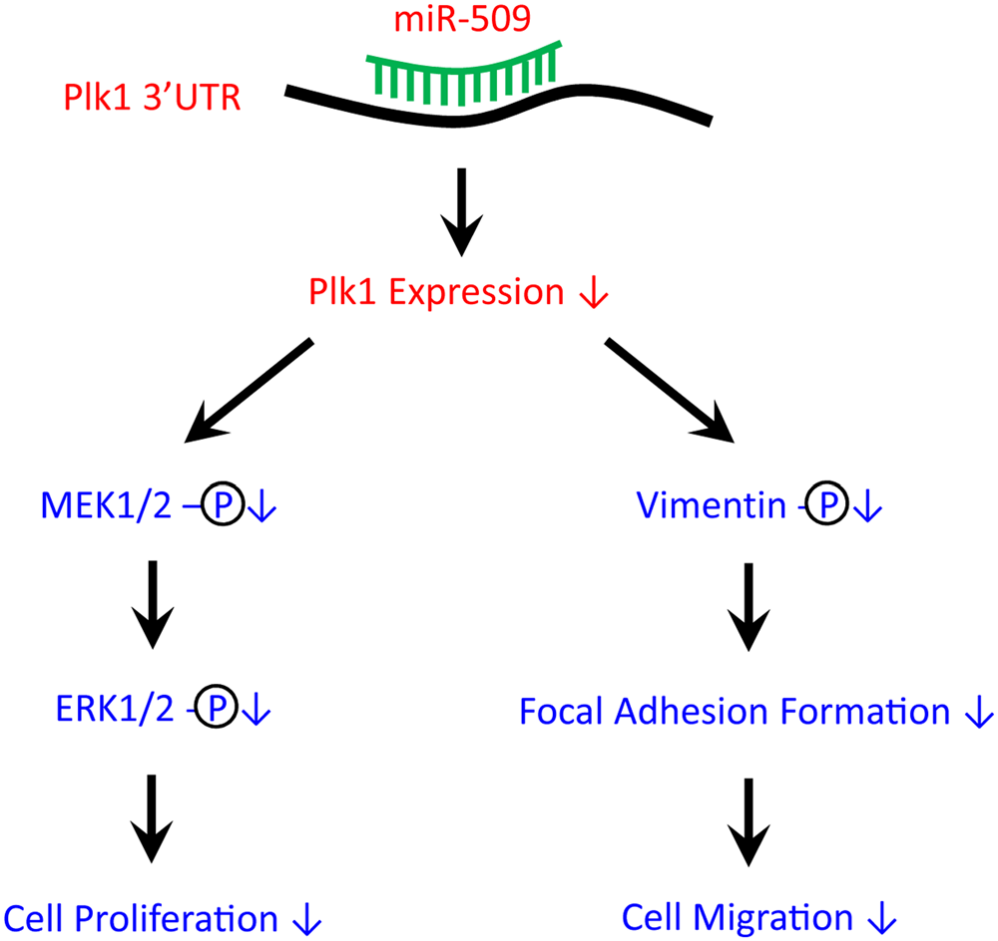
Proposed model: miR-509 targets 3′UTR of Plk1 and reduces its expression, which subsequently inhibits the growth factor-induced phosphorylation of MEK1/2 and ERK1/2, and proliferation in smooth muscle cells. Reduced Plk1 expression also diminishes vimentin phosphorylation and organization of the vimentin network, focal adhesion assembly, and migration.

In summary, we unveil a novel mechanism by which miR-509 regulates the MAPK pathway and the vimentin network. We identified miR-509 as a regulator for Plk1 expression in human airway smooth muscle cells. miR-509 regulates the MAPK pathway and cell proliferation by targeting Plk1. Moreover, miR-509 modulates reorganization of the vimentin network, focal adhesion formation, and migration through Plk1 (Fig. 6).

## Materials and Methods

### Cell Culture

Human airway smooth muscle (HASM) cells were prepared from human bronchi and adjacent tracheas obtained from the International Institute for Advanced Medicine^10,45–48^. Human tissues were non-transplantable and consented for research. This study was approved by the Albany Medical College Committee on Research Involving Human Subjects. All methods were performed in accordance with the NIH guidelines and regulations. Informed consent had been obtained from patients who donated their tracheas/bronchi. Briefly, muscle tissues were incubated for 20 min with dissociation solution [130 mM NaCl, 5 mM KCl, 1.0 mM CaCl_2_, 1.0 mM MgCl_2_, 10 mM Hepes, 0.25 mM EDTA, 10 mM D-glucose, 10 mM taurine, pH 7, 4.5 mg collagenase (type I), 10 mg papain (type IV), 1 mg/ml BSA and 1 mM dithiothreitol]. All enzymes were purchased from Sigma-Aldrich. The tissues were then washed with Hepes-buffered saline solution (composition in mM: 10 Hepes, 130 NaCl, 5 KCl, 10 glucose, 1 CaCl_2_, 1 MgCl_2_, 0.25 EDTA, 10 taurine, pH 7). The cell suspension was mixed with Ham’s F12 medium supplemented with 10% (v/v) fetal bovine serum (FBS) and antibiotics (100 units/ml penicillin, 100 µg/ml streptomycin). Cells were cultured at 37 °C in the presence of 5% CO_2_ in the same medium. The medium was changed every 3–4 days until cells reached confluence, and confluent cells were passaged with trypsin/EDTA solution^10,18,49,50^. Smooth muscle cells within passage 12 were used for the studies, which is typical for this type of cells^51,52^. Furthermore, α-actin staining, morphology, proliferation rate and cell size were not significantly different between *passage* 3 and *passage* 12 (Fig. S2). Primary cells from three non-asthmatic donors were used for most experiments. In some cases, duplicate or triplicate experiments from cells of one donor were used for analysis. Moreover, primary cells from five non-asthmatic donors and four asthmatic donors were used to compare biological properties of non-asthmatic cells and asthmatic cells.

#### Assessment of mRNA Expression

Total RNA was isolated by using the High Pure RNA Isolation Kit (Roche). The levels of mRNA were determined by reverse transcription quantitative real-time PCR (RT-qPCR). For the detection of human Plk1 mRNA, the 5′-primer sequence was 5′-GAG GAG TAC GGC TGC TGC AAG GAG-3′; the 3′-primer sequence was 5′-GAG ACG GTT GCT GGC CGA GCG TGA-3′. Human β2-microglobulin (B2M) mRNA was used as a control. The 5′-primer sequence of B2M was 5′-TGC TGT CTC CAT GTT TGA TGT ATC T-3′; the 3′-primer sequence of B2M was 5′-TCT CTG CTC CCC ACC TCT AAG T-3′. Briefly, total RNA and primers were mixed with the iTaq Universal SYBR Green One-Step Kit (Bio-Rad) and the mRNA levels were detected using a real-time PCR detection system (Bio-Rad). The expression level of Plk1 mRNA was expressed as the ratio of Plk1 mRNA over B2M mRNA.

#### Measurement of miRNA Expression

The NCode miRNA First-Strand cDNA Synthesis Kit (Life Technologies) was used to generate poly A tailing of miRNAs from purified total RNA and the first-strand cDNA. The sequence of universal RT primer was 5′-CAG GTC CAG TTT TTT TTT TTT TTT VN-3′. Afterwards, the real-time PCR (qPCR) amplification was performed by using the SsoAdvanced Universal SYBR Green Supermix Kit (Bio-Rad). The sense sequence of human miR-509 was 5′-GCA GTA CTG CAG ACG TGG CA-3′. The antisense sequence of miR-509 was 5′-CAG GTC CAG TTT TTT TTT TTT TTT CAT GAT TG-3′. The sense sequence of U6-2 housekeeping gene was 5′-CGC TTC GGC AGC AC-3′. The antisense sequence of U6-2 was 5′-GCC ATG CTA ATC TTC TCT GTA TC-3′. All primers were purchased from Applied Biological Materials Inc (Richmond, BC, Canada). The expression level of miR-509 was expressed as the ratio of miR-509 over U6-2 RNA.

#### Cell Transfection

miR-509 (hsa-miR-509-3-5p, CAT#4464066/MIMAT0004975), miR-control (CAT#4464058), miR-100 (hsa-miR-100-5p, CAT#4464066/MIMAT0000098), miR-509 inhibitor (CAT#4464084/MIMAT0004975), and miR inhibitor negative control (CAT#4464076) were purchased from Ambion/Life Technologies. Cell transfection was performed by using the lipofectamine 2000 reagent (Invitrogen) according to the manufacturer’s manual.

### Lentivirus-mediated Plk1 Expression

Plk1 cDNA was cut off from pcDNA3 3xFlag-Plk1^7^ and subcloned to pLenti-puro (Addgene #39481) at XhoI and ApaI sites. pLenti-puro has 3′UTR and poly A of BGH, which does not have miR-509 targeting sequence, and is resistant to miR-509. To produce viruses, 293FT cells were transfected with pLenti-puro encoding Plk1 plus packaging vector pCMV and envelop vector pVSV-G. Viruses were collected 48 h after transfection. For infection, smooth muscle cells were incubated with viruses 12 h. They were then cultured in the F12 growth medium for 3 days. Positive clones were selected by puromycin. Stable Plk1 expressing cells were treated with miR-509 to generate Plk1 rescue cells.

#### Assessment of Cell Proliferation

Cells (4.8 × 10^4^) were plated in the F12 medium supplemented with 10% FBS (Invitrogen) for ≥18 hours. For some experiments, cells were then transfected with miR-509 mimic or miR-control. They were subsequently treated with human platelet-derived growth factor (PDGF)-BB (Sigma, 10 ng/ml) in the F12 medium containing 0.25% FBS. Additional cells were cultured in the medium with 0.25% FBS as a control. Numbers of viable cells were evaluated using the trypan blue exclusion test. Triplicated samples were averaged for each experiment.

### Immunoblot Analysis

Cells were lysed in SDS sample buffer composed of 1.5% dithiothreitol, 2% SDS, 80 mM Tris-HCl (pH 6.8), 10% glycerol and 0.01% bromophenol blue. The lysates were boiled in the buffer for 5 min and separated by SDS-PAGE. Proteins were transferred to nitrocellulose membranes. The membranes were blocked with bovine serum albumin or milk for 1 h and probed with use of primary antibodies followed by horseradish peroxidase-conjugated secondary antibodies (Fisher Scientific). Proteins were visualized by enhanced chemiluminescence (Fisher Scientific) using the GE Imager 600 System. Antibody used were anti-Plk-1 (Millipore, Cat#05-844, Lot#2477015), anti-c-Abl (Cell Signaling, Cat#2862 S, Lot#13), anti-paxillin (BD Biosciences, Cat#610051, Lot#7208686), anti-GAPDH (glyceraldehyde 3-phosphate dehydrogenase. Ambion, Cat# AM4300, Lot#1311029), anti-MEK1/2 (Santa Cruz, Cat#Sc-436, Lot# H3011), anti-ERK1/2 (Cell Signaling, Cat#4695, Lot#8), anti-p-MEK1/2 (Santa Cruz, Cat# Sc-81503, Lot# I1813), anti-p-ERK1/2 (Cell Signaling, Cat# 9106 S, Lot# 38). Antibodies against phospho-vimentin (Ser-56) and total vimentin were custom made by Synpep Inc (CA, USA) and previously characterized^18,36^. The levels of proteins were quantified by scanning densitometry of immunoblots (Fuji Multigauge Software). The luminescent signals from all immunoblots were within the linear range.

### Luciferase Activity Analysis

The reporter construct for Plk1 3′UTR (p-EZX-MT01) was purchased from GeneCopoeia (Maryland, USA). Mutant Plk1 3′UTR reporter was generated by using Quick Change II XL Site-directed Mutagenesis Kit (Agilent Technologies) as previously described^7,19^. The sequence of the forward primer was 5′-GGCTCCCGCGGTGCCATGAGAGGTCTGTGCCCCCCAGCCCCGG-3′. The sequence of the reverse primer was 5′-CCGGGGCTGGGGGGCACAGACCT CTCATGGCACCGCGGGAGCC-3′. Following bacterial transformation, the constructs were purified by using the QIAprep Spin Miniprep Kit (Qiagen). DNA sequencing was performed by Genewiz.

To assess the luciferase activity, HEK 293 cells were transfected with wild type or mutant Plk1 3′UTR reporter plus miR-control or miR-509 for 2 days. The luciferase activity was evaluated using a microplate reader (Glomax Multidetection system, Promega).

### Time-lapse Microscopy

Cell motility was evaluated by time-lapse microscopy. Cells were plated in 6-well culture dishes with Ham’s F12 medium supplemented with 10% FBS until 20–30% of confluence. Culture dishes were then placed in a chamber at 37 °C and filled with 5%CO_2_. Cell migration was recorded live every 10 min for 16 h using a Leica DMI600 microscope system. A 10x/dry phase-contrast objective was used for image capture. The NIH ImageJ software was used to quantitatively assess net distance, total distance, velocity and directionality.

### Immunofluorescent Analysis

Cells in dishes containing coverslips were fixed for 15 min in 4% paraformaldehyde, and were then washed three times in phosphate-buffered saline (PBS) followed by permeabilization with 0.2% Triton X-100 dissolved in PBS for 5 min. These cells were immunofluorescently stained using specific antibodies followed by appropriate secondary antibodies (Invitrogen). Z-stack images of labeled proteins were taken on the Zeiss LSM880 microscope with Airyscan, and analyzed using the Imaris software (Bitplane). Vimentin filaments were 3D-rendered using the FilamentTracer application on Imaris. Paxillin focal adhesions were 3D-rendered using the surface algorithm on Imaris. To quantify the percentage of vimentin filaments contacting paxillin surfaces, a mask was applied to the 3D-rendered vimentin filaments to generate a new channel based on intensity. Next, we applied a surface render to the new vimentin channel, where we performed a distance transformation on vimentin so that any paxillin surface that contacts a filament had an intensity value of zero, while anything not contacting a filament had a value greater than zero. We then filtered the paxillin channel based on the distance transformation to obtain the number of surfaces contacting filaments and the number of surfaces not contacting filaments. There we obtained a percentage of vimentin filaments contacting paxillin surfaces based on our treatments. The remaining filament and surface parameters were calculated during rendering and exported from Imaris to GraphPad for statistical analysis^45,46,48,53^.

### Statistical Analysis

All statistical analysis was performed using Prism software (GraphPad Software, San Diego, CA). Differences between pairs of groups were analyzed by Student’s *t*-test. Comparison among multiple groups was performed by one-way or two-way ANOVA followed by a post hoc test (Tukey’s multiple comparisons). Values of n refer to the number of experiments used to obtain each value. P < 0.05 was considered to be significant.

## Electronic supplementary material

Supplementary Information

## Data Availability

All data generated or analyzed during this study are included in this published article. All research materials are available upon request by qualified investigators.
