# Supplementary material for: MicroRNA miR-509 Regulates ERK1/2, the Vimentin Network, and Focal Adhesions by Targeting Plk1
Source: Sci Rep. 2018 Aug 22;8:12635. doi: 10.1038/s41598-018-30895-8 (PMC6105636; doi:10.1038/s41598-018-30895-8)
Supplement: Supplementary file 1 — Supplementary Information [file 41598_2018_30895_MOESM1_ESM.pdf]

Supplementary Figure

MicroRNA miR-509 Regulates ERK1/2, the Vimentin Network, and Focal Adhesions by Targeting Plk1

Guoning Liao, Ruping Wang, Alyssa C. Rezey, Brennan D. Gerlach, and Dale D. Tang*

Department of Molecular and Cellular Physiology

Albany Medical College, Albany, New York, U.S.A.

*To whom correspondence should be addressed: Dale D. Tang, Department of Molecular and Cellular Physiology, Albany Medical College, 47 New Scotland Avenue, MC-8, Albany, NY 12208, Tel: (518)-262-6416; Fax: (518)-262-8101; E-mail: tangd@amc.edu

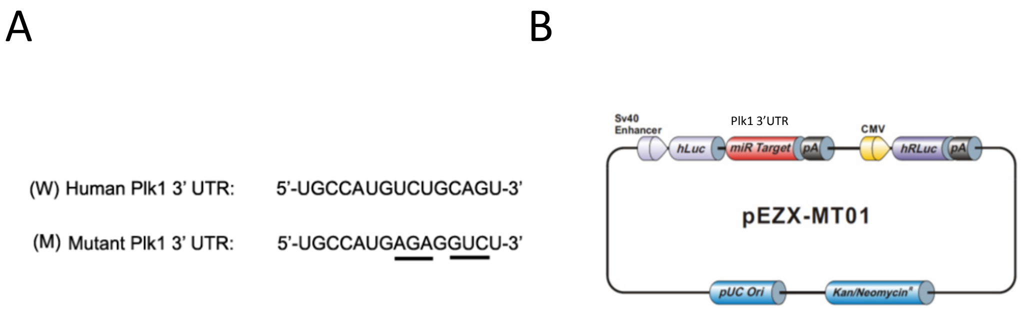

Figure S1. A. Mutagenesis of Plk1 3' UTR. Shown are the 6 mutated bases in the miR-509 targeting sequence of the 3'UTR of Plk1. **B. Schematic illustration of Plk1 3'UTR reporter.** The reporter uses a dual-reporter system, which uses firefly luciferase as the 3'UTR reporter and renilla luciferase as the internal control for signal normalization (GeneCopoeia).

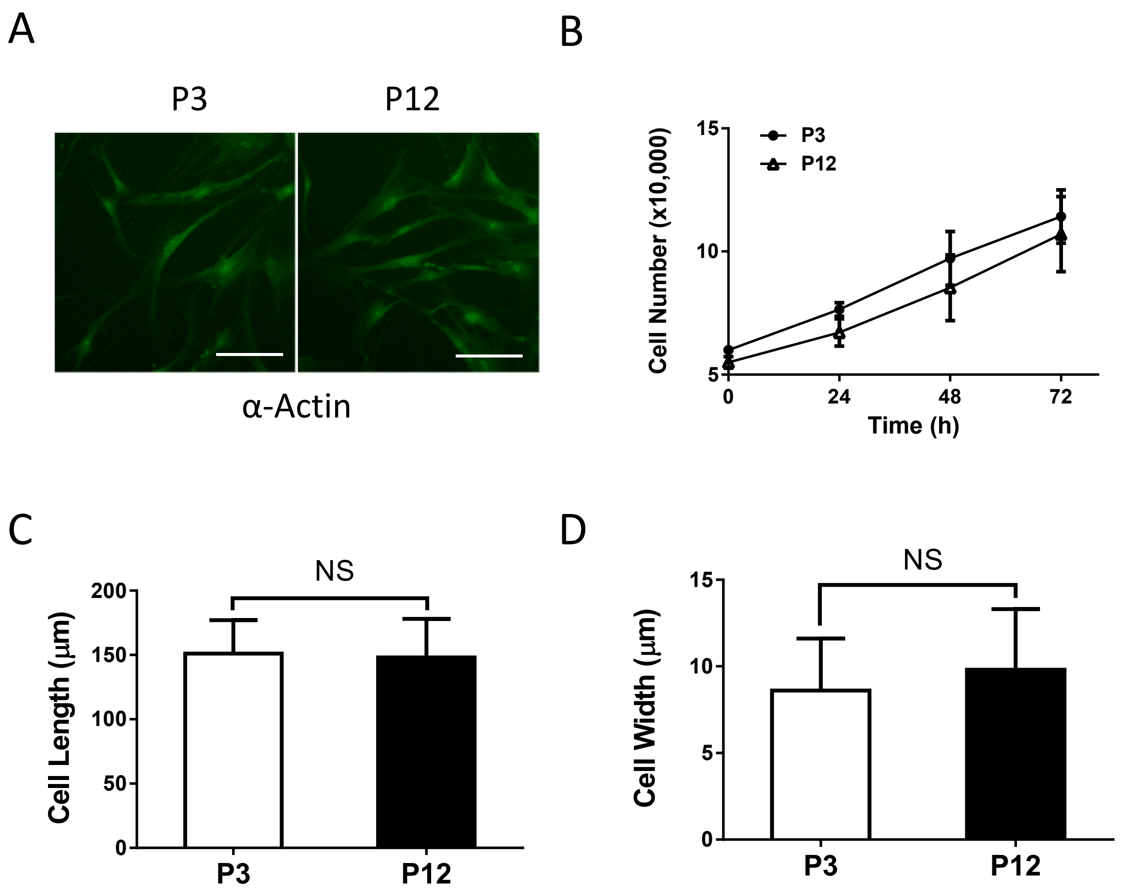

Figure S2. Characterization of low and high passage human airway smooth muscle cells. A. Passage 3 (P3) and Passage 12 (P12) human airway smooth muscle cells were immunostained for α-actin. α-Actin fluorescence is similar between P3 and P12 cells. Most cells are long and spindle-like shape. Scar bar, 50 μm. **B.** Proliferation rate of P3 and P12 cells is not significantly different upon PDGF activation ($n = 4$, $P > 0.05$). **C.** Length of P3 and P12 cells is not significantly different ($n = 115$, $P > 0.05$). **D.** Width of P3 and P12 cells is not significantly different ($n = 115$, $P > 0.05$). NS, not significant. Student's *t*-test was used for statistical analysis.

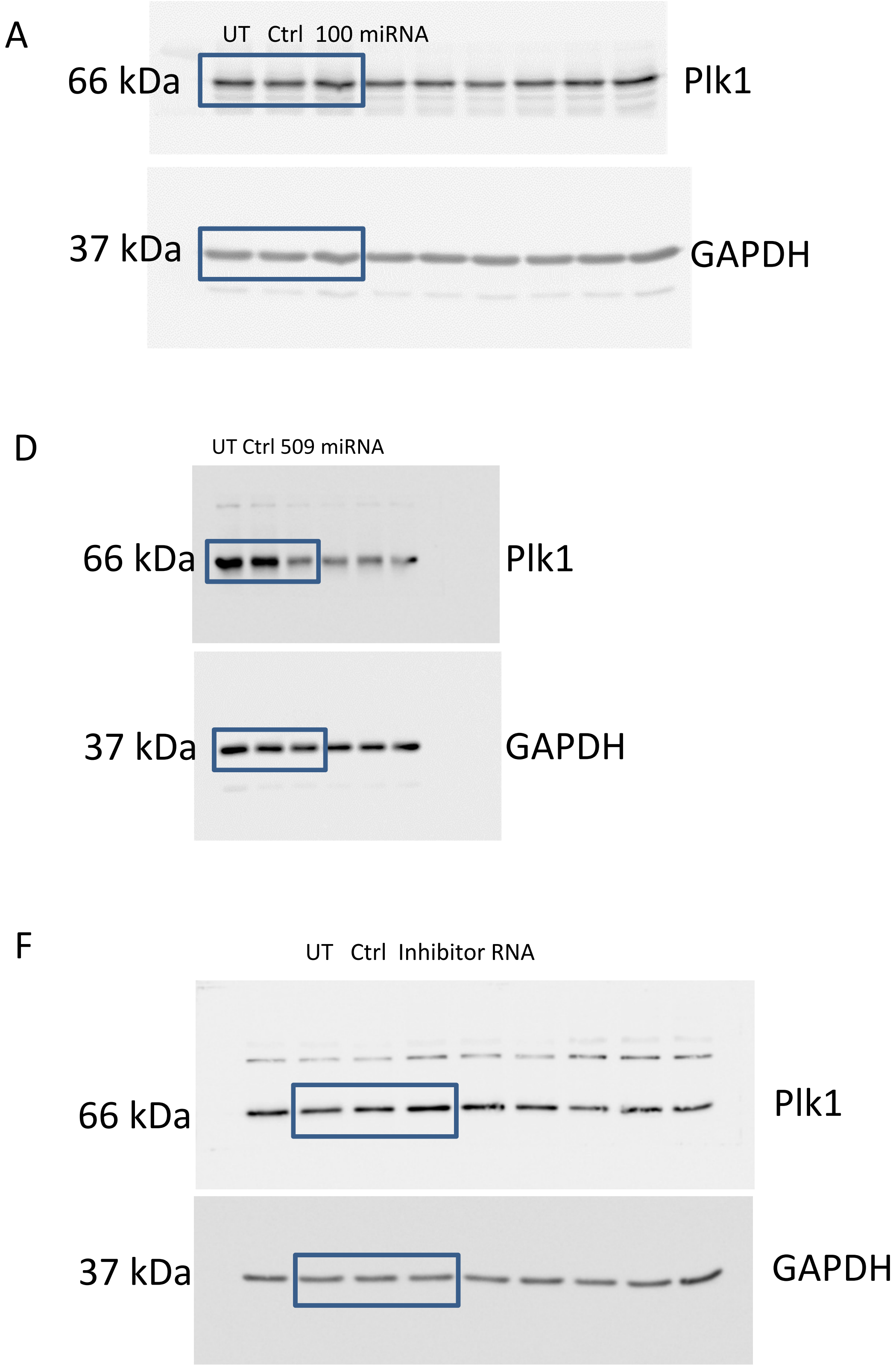

Figure S3. Original blots for Figure 1.

Figure 2C

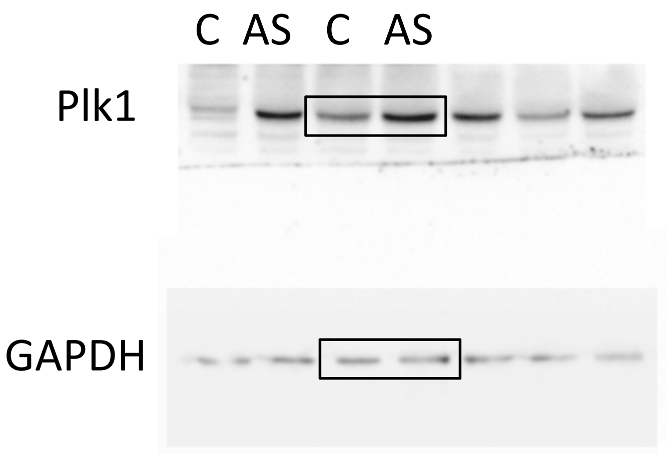

Figure 3B

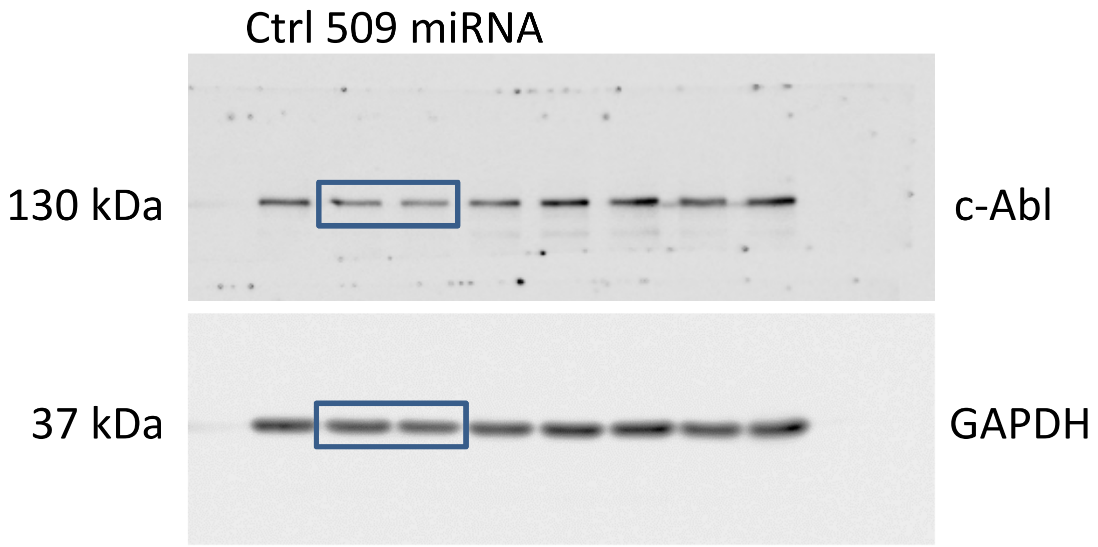

Figure S4. Original blots for Figure 2C and Figure 3B.

Figure 3C

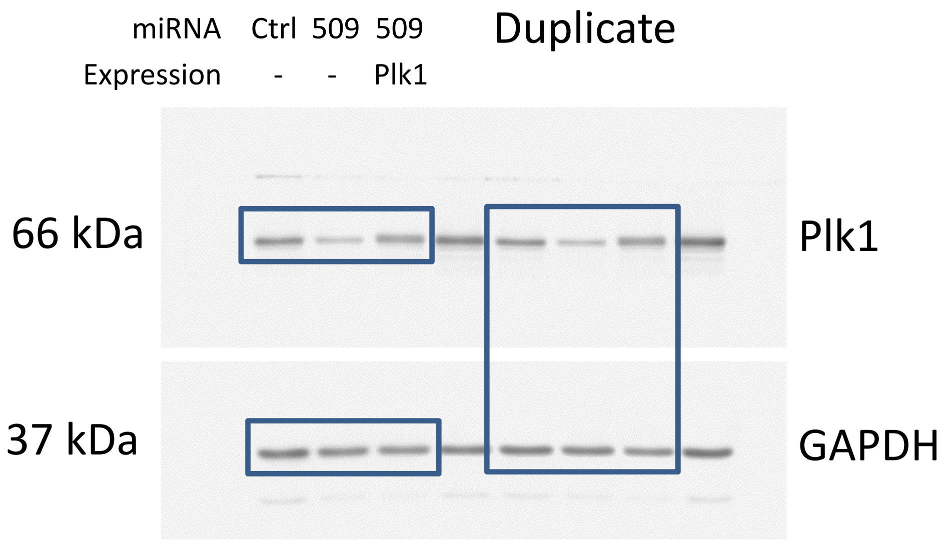

Figure 3D

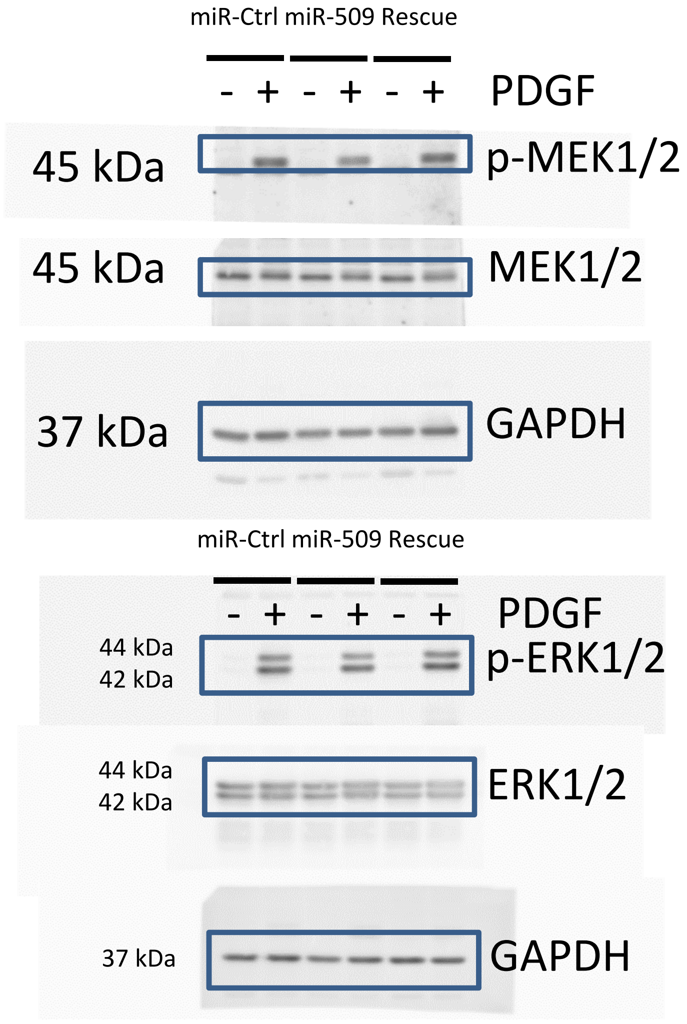

Figure S5. Original blots for Figure 3C and 3D.

Figure 4F

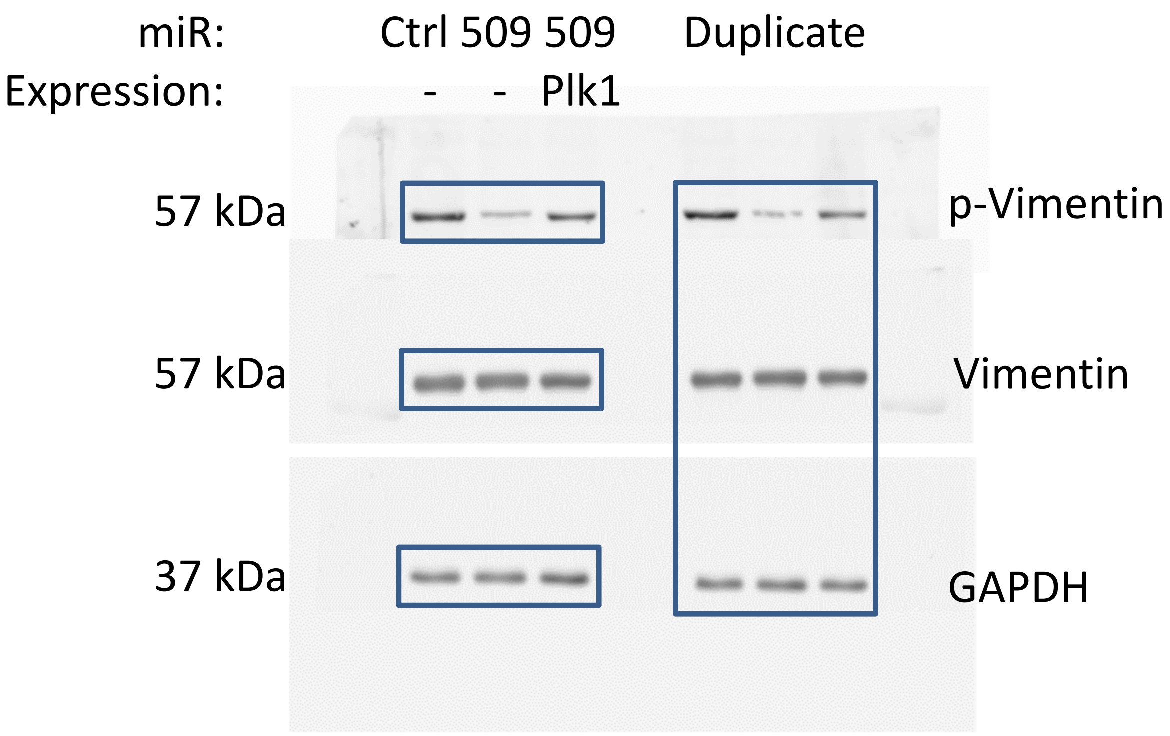

Figure S6. Original blots for Figure 4F.
